# Genetic structure, spatial organization, and dispersal in two populations of bat-eared foxes

**DOI:** 10.1002/ece3.683

**Published:** 2013-07-26

**Authors:** Jan F Kamler, Melissa M Gray, Annie Oh, David W Macdonald

**Affiliations:** 1 Wildlife Conservation Research Unit, Department of Zoology, The Recanati-Kaplan Centre, University of OxfordTubney House, Abingdon Road, Tubney, Abingdon, OX13 5QL, UK; 2 Department of Ecology and Evolutionary Biology, University of California-Los AngelesLos Angeles, California, 90095

**Keywords:** Density, group size, home-range overlap, *Otocyon megalotis*, philopatry, South Africa

## Abstract

We incorporated radio-telemetry data with genetic analysis of bat-eared foxes (*Otocyon megalotis*) from individuals in 32 different groups to examine relatedness and spatial organization in two populations in South Africa that differed in density, home-range sizes, and group sizes. Kin clustering occurred only for female dyads in the high-density population. Relatedness was negatively correlated with distance only for female dyads in the high-density population, and for male and mixed-sex dyads in the low-density population. Home-range overlap of neighboring female dyads was significantly greater in the high compared to low-density population, whereas overlap within other dyads was similar between populations. Amount of home-range overlap between neighbors was positively correlated with genetic relatedness for all dyad-site combinations, except for female and male dyads in the low-density population. Foxes from all age and sex classes dispersed, although females (mostly adults) dispersed farther than males. Yearlings dispersed later in the high-density population, and overall exhibited a male-biased dispersal pattern. Our results indicated that genetic structure within populations of bat-eared foxes was sex-biased, and was interrelated to density and group sizes, as well as sex-biases in philopatry and dispersal distances. We conclude that a combination of male-biased dispersal rates, adult dispersals, and sex-biased dispersal distances likely helped to facilitate inbreeding avoidance in this evolutionarily unique species of Canidae.

## Introduction

The genetic structure within populations has been shown to be affected by dispersal and philopatry patterns among birds and mammals (Loison et al. 1999; Ji et al. 2001; Temple et al. 2006; Costello et al. 2008; Ortego et al. 2008). Determining the genetic structure in mammals also has been useful for explaining cooperation (Widdig et al. 2001; Creel and Creel 2002), space use (Støen et al. 2005; Maher 2009), mating systems (Dugdale et al. 2008; Wright et al. 2010), prey use (Carmichael et al. 2001), dispersal distances (Spong and Creel 2001), and habitat use (Sacks et al. 2005) within populations. Among terrestrial carnivores, both solitary and group living species have exhibited genetic clustering of relatives based on female philopatry and male dispersal. For solitary carnivores, establishment of breeding ranges of females within or adjacent to their natal ranges resulted in genetic clustering of females in populations of black bears (*Ursus americanus*; Costello et al. 2008), brown bears (*U. arctos*; Støen et al. 2005), raccoons (*Procyon lotor*; Ratnayeke et al. 2002), and bobcats (*Lynx rufus*; Janecka et al. 2006). For group living carnivores, recruitment of young females into their natal groups resulted in higher female relatedness within compared to between groups for white-nosed coatis (*Nasua narica*; Gompper et al. 1998), lions (*Panthera leo*; Spong et al. 2002) and spotted hyenas (Van Horn et al. 2004). Striped hyenas (*Hyaena hyaena*) are an exception to the above trends, as this species did not exhibit genetic clustering of either sex, due to the tendency of both sexes to disperse farther than neighboring ranges (Wagner et al. 2007). The primary reason for male-biased dispersal in mammals, and consequently genetic clustering of philopatric females, appears to be inbreeding avoidance (Pusey 1987; Wolff 1994; Costello et al. 2008), although other factors also may be involved (Moore and Ali 1984; Dobson and Jones 1985).

Canids are unique among carnivores in that monogamy, long-term pair formation, and male parental care are characteristic of species in this group (Kleiman and Eisenberg 1973). In contrast to most carnivores, the unique behavioral characteristics of canids have not resulted in consistent genetic clustering of females within populations, although genetic structures varied considerably within and among canid species. Among small (<5 kg) canids that consist primarily of mated pairs, genetic clustering of females occurred in some species (Ralls et al. 2001; Kitchen et al. 2005) but not others (Roemer et al. 2001). Among large (>10 kg) canids that often live in packs, genetic clustering of relatives tended to occur among neighboring groups, although intersexual differences were not consistent across studies for gray wolves (*Canis lupus*; Lehman et al. 1992), Ethiopian wolves (*C. simensis*; Randall et al. 2007), coyotes (*C. latrans*; Williams et al. 2003), and African wild dogs (*Lycaon pictus*; Girman et al. 1997). Clearly, genetic structure and clustering within canid populations show large variation, which may be dependent on pair versus group living, dispersal distances, sex-biased philopatry, and mortality. That said, the effects of density and group size on the genetic structure within populations of canids in particular, and carnivores in general, are not well understood and have received little attention (Lehman et al. 1992).

The bat-eared fox (*Otocyon megalotis*) is a small canid in Africa that is often regarded as a group living species, although some populations consist primarily of mated pairs (Lamprecht 1979; Nel et al. 1984; Malcolm 1986; Maas and Macdonald 2004; Wright et al. 2010). Bat-eared foxes are not considered highly territorial, as group ranges appear to overlap significantly with little aggression between groups (Koop and Velimirov 1982; Nel 1993). Reasons for pair living versus group living in bat-eared foxes are not known, but are thought to be related to mortality, predation levels, abundance and dispersion of food resources (Nel et al. 1984; Maas and Macdonald 2004; Kamler et al. 2012, 2013). Group formation of bat-eared foxes, when it occurs, was thought to result from female offspring recruitment into natal groups (Maas and Macdonald 2004). However, young males also were sometimes reported to remain within their natal ranges beyond the next breeding season (Malcolm 1986; Maas and Macdonald 2004), thus it is not clear if female or male philopatry is primarily responsible for group living in this species. Also, young bat-eared foxes were reported to establish home ranges and breed in areas adjacent to their natal ranges (Maas and Macdonald 2004), which could result in clustering of relatives for both sexes. Because the genetic structure of bat-eared fox populations has not been studied, it is not known to what extent pair living versus group living, sex-biased philopatry, and dispersal patterns affects spatial and genetic structure. Additionally, the apparent tolerance of neighboring groups of bat-eared foxes might be associated with genetic relatedness, similar to that reported for swift foxes (Kitchen et al. 2005) and other carnivore species (Gompper et al. 1998; Ratnayeke et al. 2002; Moyer et al. 2006).

We studied genetic and spatial structure of two subpopulations of bat-eared foxes in South Africa. The subpopulations occurred on different properties separated by 10 km with several additional fenced properties in between, and densities and home-range sizes differed between sites. Importantly, the lower density population consisted primarily of mated pairs whereas the higher density population consisted primarily of groups. Thus, we could examine if such demographic differences affected the genetic and spatial structuring within subpopulations. Therefore, we evaluated spatial and genetic structure of male, female, and mixed-sex dyads, and compared results to patterns of philopatry and dispersal distances of both sexes. Furthermore, we examined whether home-range overlap was associated with genetic relatedness. We hypothesized that (1) at a fine scale, genetic clustering would occur in both populations for females, but not males, due to female philopatry; (2) at a broad scale, distances between foxes would be correlated with relatedness in both populations for both sexes; (3) home-range overlap would be correlated with relatedness for both sexes in both populations, and; (4) philopatry and dispersal distances would be consistent with patterns of genetic structure for both sexes.

## Materials and Methods

This article was part of a larger study investigating the ecology of bat-eared foxes and other canids on two study sites in South Africa (Kamler et al. 2012, 2013). One of the main goals of the larger study was to determine if differences in black-backed jackal (*Canis mesomelas*) density affected the ecology of bat-eared foxes (Kamler et al. 2013). Capture, radio-telemetry monitoring, and observations at den sites were the primary methods used to collect data on individual foxes and their respective groups. Collection and subsequent analyses of DNA samples allowed us to conduct post hoc investigations of relatedness within subpopulations, and to compare results to spatial and dispersal patterns. It is important to emphasize that bat-eared foxes were numerous, living at relatively high densities on both sites (see below), and that our efforts were focused on capturing and sampling 1–2 foxes/group from as many adjacent groups as possible, rather than capturing and sampling all or most foxes within fewer groups.

### Study sites and animal capturing

The research was conducted on two sites near Kimberley, South Africa. Site 1 was on Benfontein Game Farm (hereafter Benfontein; 110 km^2^; 28°53′ S, 24°49′ E) located 8 km southeast of Kimberley, and site 2 was on private ranches (PR; 81 km^2^; 28°59′S, 24°48′E) located 5 km south of Benfontein. The area contained elements of three major biomes, Savanna, Nama Karoo, and Grassland, although Nama Karoo vegetation dominated (>65%) both sites. The area has a semiarid continental climate, with a distinct cold and dry period during winter (March–August), a hot and rainy period during summer (September–February), and a mean (±SD) annual rainfall of 419 ± 134 mm (Kamler et al. 2012). Both sites were managed for wild ungulates and livestock. Numbers of black-backed jackals, a predator of bat-eared foxes, were relatively high on Benfontein, but relatively low on PR (Kamler et al. 2013). More detailed descriptions of the study sites and species present are provided by Kamler et al. (2012, 2013).

From August 2005 to March 2008, we captured, radio-collared, and monitored 23 and 18 bat-eared foxes on Benfontein and PR, respectively. Foxes were captured using wire box traps (50 × 50 × 120 cm) baited with meat scraps, which were placed along dirt roads and intersections throughout the study site, with >0.5 km separating each trap (see Kamler et al. 2012 for more details of trapping procedures). We fitted captured foxes with radio collars weighing 1–2% of their body mass. All foxes were sexed, weighed to the nearest 0.1 kg, and classified as adult (≥24 months old), yearling (12–23 months old), and juvenile (<12 months old) based on tooth wear, body size, and reproductive condition, then released immediately at the capture site. Additionally, one lower canine tooth was pulled from the skulls of five bat-eared that died during the study to count cementum annuli and to confirm ages (Kamler and Macdonald 2006). Adult females were considered breeders if they showed signs of nursing (i.e., dark and elongated teats) during or after the pup-rearing season. Adult males were considered breeders if they were closely associated with adult females during the breeding season, and were closely associated with pups during the pup-rearing periods. For aging purposes, we assumed a birth date of 1 November, which was consistent with births on our study sites and in other areas of southern Africa (Smithers 1971; Nel et al. 1984). Our research and handling protocol was approved by the Department of Tourism, Environment and Conservation, Kimberley, South Africa, and followed the animal care and use guidelines of the American Society of Mammalogists (Gannon et al. 2007).

### Microsatellite DNA analysis

We collected hair samples to obtain microsatellite genotypes from 19 foxes on Benfontein, and 16 foxes on PR. Collected samples were hairs with fresh follicles (*n* = 10–15/fox) that were pulled from the hind leg of captured foxes, and stored in plastic tubes containing 90% ethanol. Genomic DNA was extracted from tissue samples using the QiaAmp DNA blood mini kit (Qiagen, Hilden, Germany). Individuals were genotyped using five dinucleotide (Goldstein et al. 1999), and seven tetra-nucleotide fluorescently labeled microsatellite markers (Guyon et al. 2003). All markers were located on different chromosomes or separated by large >15 Mb. One labeled and one unlabeled primer (20 pmol) were added to 50 ng genomic DNA, 0.2 mmol/L dNTP, 2.5 mmol/L MgCl_2_, 1× DNA reaction buffer, and 0.8 units of Taq DNA polymerase (Promega, Madison, WI) in a reaction volume of 25 μL. The polymerase chain reaction (PCR) amplification was performed on an MJ Research PTC 100 Thermal Cycler under the following conditions: 94°C for 5 min, 30 cycles at 94°C for 45 sec, 54–62°C for 45 sec, and 72°C for 1 min, and a final extension at 72°C for 5 min. The PCR products were then run on an ABI3700 (capillary system) sequencer (Applied Biosystems Inc., CA) and genotyped using the GENEMAPPER analysis software (Applied Biosystems Inc., CA).

General population statistics such as allele frequency, Hardy–Weinberg equilibrium, heterozygosity, *F*_ST_, and *R*_ST_ were calculated in Genepop (Raymond and Rousset 1995; Rousset 2008). Principal coordinate analysis (PCA) was performed with the GenAlEx add-on software (Peakall and Smouse 2006) in Excel. To determine relatedness at the sibling or parental level between individuals, the program KINSHIP 1.2 (Queller and Goodnight 1989) was used to calculate an index of relatedness (*r*) for all possible dyads of individuals. The two study sites were analyzed separately. To assess the significance of observed *r-*values we simulated 1000 replicates of *r-*values for full sibs (*r* = 0.5) and unrelated individuals (*r* = 0) using KINSHIP. The observed *r-*values were then compared to the frequency distributions of simulated related and unrelated individuals to determine if pairs fell in the “unrelated” or “related” category. Cutoff values were determined by using two standard deviations from the mean of the simulated “unrelated” and “related” *r-*values. Thus, any pair with an *r-*value below two standard deviations from the related mean value was considered “unrelated,” any pair with an *r*-value above the two standard deviations from the unrelated mean value was considered “related,” whereas any pair with an *r*-value between two standard deviations from both mean values was “unsure.” We chose two standard deviations as the cutoff because it captured 95% of the data for each distribution. There was no overlap between these two cutoff points, it left few “unsure” data points, and overall was a relatively easy method to group samples based on relatedness. The distributions of simulations of related and unrelated individuals are given in Figure A1.

### Spatial and statistical analyses

Radio-telemetry methods, densities, mean group sizes, and mean annual home-range sizes of bat-eared foxes on both were reported in previous papers (Kamler et al. 2012, 2013). In summary, adult densities were 1.07 fox/km^2^ on Benfontein, and 0.68 fox/km^2^ on PR. Mean adult group size was significantly larger on Benfontein (4.37 ± 0.30 fox/group) than on PR (2.28 ± 0.24 fox/group), and mean annual home-range size was significantly larger on Benfontein (4.96 ± 0.32 km^2^) than on PR (2.79 ± 0.30 km^2^).

In this article, we considered home ranges and spatial associations only of foxes for which DNA samples were obtained. Unless otherwise noted, all foxes mentioned hereafter had their DNA analyzed successfully. Home ranges were calculated for foxes in this study if we collected >20 locations and monitored ≥4 months, although most (77%) foxes had >30 locations and >9 months of monitoring. Area observation curves, which plotted home-range size against number of locations, showed that the minimum number of locations was obtained to effectively determine home-range sizes for all individual foxes used in this study. Home ranges were calculated using the 96% minimum convex polygon method (Kamler et al. 2012, 2013). Multiple home ranges were calculated for foxes monitored >1 year, however, only one home range was used in analyses. The genetic and spatial data were compared only between foxes that were monitored simultaneously for >1 month. Foxes were considered to belong to the same group if they were located together >50% of the time for which they were monitored. Across both sites, monitored foxes represented individuals captured as breeding adults (*n* = 18), individuals captured as juveniles or yearlings but that later became breeding adults within the study sites (*n* = 5), and individuals captured as juveniles or yearlings that later died (*n* = 1) or dispersed off the study sites (*n* = 9). Two yearlings that dispersed before home ranges were calculated or groups could be assigned were not used in analyses. Data from nonbreeding yearlings were used in analyses because we confirmed that all were associated with a family group that included breeding adults, at least prior to dispersal or death, thus all comparisons were among reproducing groups of bat-eared foxes. If young foxes dispersed and later bred in a different range within the study sites, we only included data from the new range in which they reproduced.

We classified foxes as neighbors if they were resident in adjacent home ranges that had some overlap, or had home-range borders separated by <100 m. To determine if kin were spatially clustered at a fine scale, we compared mean *r*-values between neighbors and non neighbors for the different sex dyads (i.e., female–female, male–male, and female–male) on each site using Mann–Whitney *U* tests. We also used an analysis of variance (ANOVA) with site, neighbor status (nested within site), and sex (nested within neighbor status) to determine which factor contributed most to differences in *r*-values.

We evaluated whether distance between home-range centroids was correlated with *r*-values to determine if genetic structure occurred at a broad scale. We computed Spearman's rank correlations between *r*-values and distance between home-range centroids for all sex dyads on both sites. Because of the issue of pseudoreplication when using multiple comparisons of relatedness from the same individual, we used Mantel's randomization tests (Mantel 1967) with 10,000 permutations to test the significance of the estimated correlations.

We determined the level of home-range overlap between neighbors for the different sex groupings, and whether *r*-values of neighbors were correlated with level of overlap. Overlap of home ranges was calculated by overlaying the polygons of two foxes and calculating the area of overlap in ArcView 3.2 (Environmental Systems Research Institute, Inc., Redlands, CA). Percent overlap was determined by multiplying the overlap area by 2, and then dividing by the total area of both polygons. We computed Spearman's rank correlations, with 10,000 permutations, between *r*-values and percent of home-range overlap for all sex dyads on both sites.

Because dispersal data were low per site, we used data from all radio-collared foxes and pooled data from sites to examine differences in dispersal patterns among age and sex groups. Dispersal distances were calculated for foxes based on distances between centroids of initial home ranges and the newly established home ranges. For foxes that dispersed off the study sites, distances were calculated from initial home-range centroids to the locations at which they were later found. The dispersal date was assumed to be the median between the last location within their initial home range and first location outside their home range or the first time that contact was lost. If contact was lost for foxes, we assumed they dispersed if we never visually observed them on the study sites again, and if collars were not sounding impaired prior to loss of contact. Of 11 foxes whose contact was initially lost, one was returned dead by a landowner outside the study site, two were located alive outside the study site using aerial telemetry from a two-person microlight, two returned briefly after several months before dispersing again (i.e., contact was lost again), and three made several excursions from their home ranges in the 2 weeks prior to lost contact (i.e., predispersal forays). For yearlings, age of dispersal was calculated as the difference from their dispersal date and assumed birth date, and was compared between sites using a Wilcoxon ranked-sum test. Dispersal rates were calculated and compared between age and sex classes using Fisher's exact tests, and foxes were only included in analyses if they lived through at least one reproductive season after initial capture. To determine if there were intersexual differences in dispersal distances, we compared (based on distance between centroids) settlements ≤5 km from initial ranges to settlements >5 km from initial ranges of both sexes using a Fisher's exact test. We chose the 5-km distance as a cutoff for analysis because foxes that dispersed within this distance settled in neighboring ranges, as well as neighboring ranges twice removed, whereas all other dispersing foxes settled in ranges that were at least three home ranges from their initial range. Additionally, we could always confirm if foxes dispersed <5 km from their initial range, but otherwise we could not confirm the dispersal distance for some foxes that dispersed >5 km. For foxes with known dispersal distances, mean dispersal distances were compared between sexes and sites using Wilcoxon ranked-sum tests.

Unless otherwise noted, statistical analyses were performed using the program SPSS 18.0 (IBM Corp., Armonk, NY), and differences were deemed significant when *P* < 0.05, and marginally significant when *P* < 0.10.

## Results

**Figure 1:**
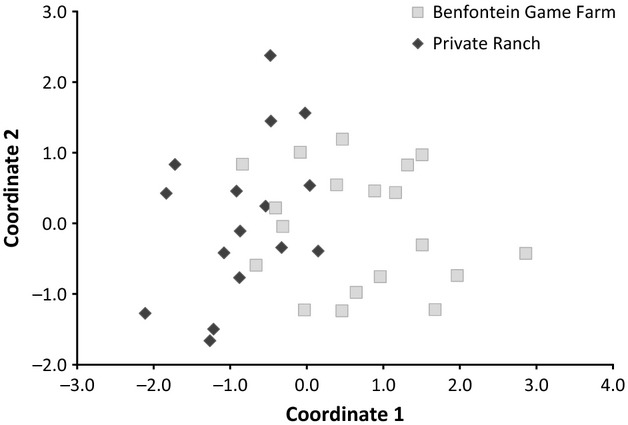
Principle coordinate analysis of 12 microsatellite markers of bat-eared foxes (*Otocyon megalotis*) monitored on two study sites in South Africa. Pairwise genetic distance of squared differences was calculated between all individuals across microsatellite loci.

The average number of loci per locus and observed heterozygosities were similar between the Benfontein and PR populations (Table 1). Additionally, similar alleles were observed between the two populations (Table 1) but F_ST_ (average across loci = 0.0675), *R*_ST_ (average across loci = 0.0279)_,_ and PCA suggested that the Benfontein and PR populations were somewhat differentiated (Fig. 1). From the KINSHIP simulation data, we observed an overlap between related and unrelated categories, thus we determined the lower cutoff value for individuals to be considered related at *r* < 0.299 for the Benfontein population and *r* < 0.316 for the PR population. The upper cutoff value for pairs to be considered unrelated was 0.208 for the Benfontein population and 0.191 for the PR population.

**Table 1:** Characteristics of microsatellite loci used to estimate relatedness in bat-eared foxes (*Otocyon megalotis*) from two study sites in South Africa, 2005–2008

Locus	Benfontein Game Farm					Private Ranch				
	
	*n*	*k*	Ho	He	HWE	*n*	*k*	Ho	He	HWE
250	19	2	0.368	0.301	0.325	16	2	0.125	0.117	0.790
279	19	4	0.579	0.486	0.082	16	3	0.563	0.525	0.701
410	19	5	0.579	0.734	0.234	16	6	0.563	0.668	0.059
606	19	7	0.789	0.745	0.979	16	6	0.625	0.711	0.341
671	19	6	0.684	0.748	0.591	16	5	0.375	0.697	0.148
2140	19	5	0.737	0.723	0.045	16	10	0.875	0.830	0.679
2274	19	8	0.895	0.783	0.581	16	9	0.813	0.793	0.350
2293	19	14	1.000	0.892	0.670	16	8	0.750	0.822	0.768
2626	19	11	0.842	0.881	0.029	16	10	0.813	0.867	0.188
2670	19	6	0.684	0.752	0.166	16	4	0.500	0.502	0.687
3489	19	9	0.789	0.827	0.486	16	9	1.000	0.852	0.301
PEZ17	18	2	0.389	0.461	0.505	16	3	0.250	0.271	0.063
Mean	18.9	6.6	0.695	0.694	0.391	16.0	6.3	0.604	0.638	0.423

The table includes locus name, number of individuals typed (*n*), number of alleles (*k*), observed heterozygosity (Ho) and expected heterozygosity (He), and Hardy–Weinberg equilibrium (HWE) for 12 microsatellite loci sampled from bat-eared foxes.

**Figure 2:**
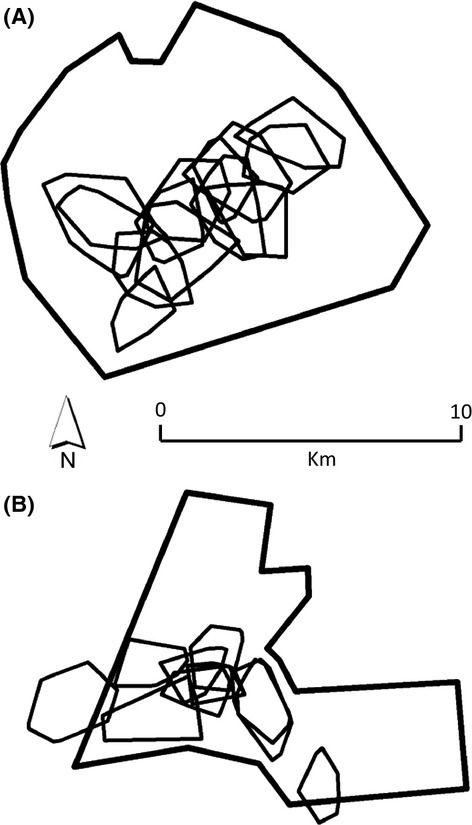
The annual home ranges of bat-eared foxes (*Otocyon megalotis*) living in groups that were simultaneously monitored on (A) Benfontein Game Farm (*n* = 13) and (B) Private Ranch (*n* = 11) in South Africa, 2006–2007. Note that additional foxes used in the analysis were monitored in other years.

The DNA was successfully extracted from samples of 19 bat-eared foxes from 18 groups on Benfontein, and 16 foxes from 14 groups on PR (Table 1; Fig. 2). Of the three fox dyads that were from the same group, two dyads were related (*r* > 0.308), and the third dyad consisted of an adult mated pair that was unrelated (*r* = 0.172). Of the two related dyads, one was a probable father–daughter dyad, whereas the other was a probable brother dyad. Incidentally, we also monitored two additional dyads that were probable brother dyads because they were highly related (*r* > 0.415), of similar age, and initially from the same group, but individuals in both dyads later split into separate groups.

**Figure 3:**
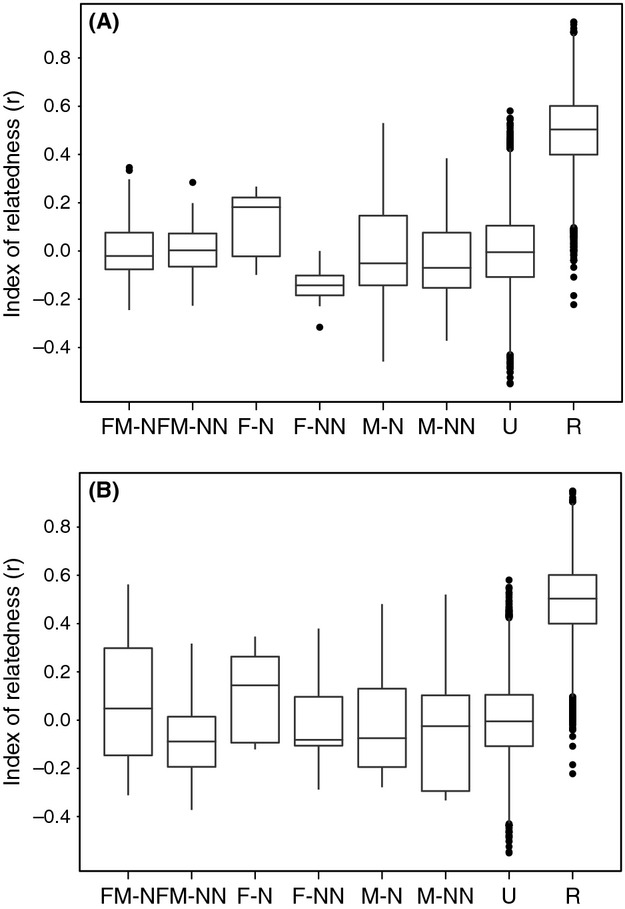
Mean (±95% CI) index of relatedness (*r*) among different dyads of bat-eared foxes (*Otocyon megalotis*) from two study sites in South Africa, 2005–2008, on (A) Benfontein and (B) Private Ranch. The figure shows results for female dyads (F), male dyads (M), and mixed-sex dyads (FM) classified as neighbor (N) or non neighbor (NN). Also shown are unrelated (U) and related (R) simulated distributions.

On Benfontein, neighbors were significantly more related than non neighbors for female dyads (*P* = 0.004), but not for male (*P* = 0.773) or mixed-sex (*P* = 0.970) dyads (Fig. 3). On PR, relatedness was similar between neighbors and non neighbors for female (*P* = 0.662), male (*P* = 0.684), and mixed-sex (*P* = 0.101) dyads (Fig. 3). The results from the ANOVA showed that neighbor status (*F* = 3.72, *P* = 0.026) was the factor with the greatest effect on *r*-values compared to site (*F* = 0.12, *P* = 0.726) and sex (*F* = 0.81, *P* = 0.592).

**Figure 4:**
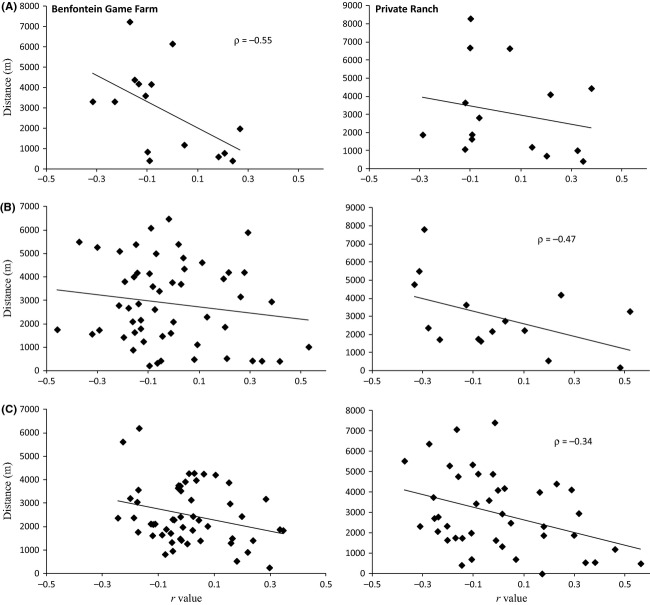
Relationship of geographic distance between home range centroids and kinship (*r*-value) in bat-eared foxes (*Otocyon megalotis*) on Benfontein (left side) and Private Ranch (right side) for (A) female dyads, (B) male dyads, and (C) mixed-sex dyads. The rho value is given for correlations that had a marginal significance (*P* < 0.10).

On Benfontein, relatedness was negatively correlated with distance for females dyads (ρ = −0.55, *P* = 0.035), but not for male (ρ = −0.11, *P* = 0.403) or mixed-sex (ρ = −0.17, *P* = 0.228) dyads (Fig. 4). On PR, there was a significant negative correlation between relatedness and distance for mixed-sex dyads (ρ = −0.34, *P* = 0.026), a marginal negative correlation for male dyads (ρ = −0.47, *P* = 0.078), but no correlation for female dyads (ρ = −0.24, *P* = 0.401, Fig. 4).

For neighboring female dyads, mean (±SE) overlap of home ranges was significantly greater (*P* = 0.038) on Benfontein (44.4 ± 7.4%) than PR (21.1 ± 6.2%). For neighboring male dyads, mean overlap of home ranges was similar (*P* = 0.606) between Benfontein (30.0 ± 5.6%) and PR (23.3 ± 13.4%). For neighboring mixed-sex dyads, mean overlap of home ranges was similar (*P* = 0.923) between Benfontein (19.8 ± 3.7%) and PR (19.1 ± 6.0%). On Benfontein, there was positive correlation between relatedness and percent home range overlap for mixed-sex dyads (ρ = 0.42, *P* = 0.019), but not for female (ρ = 0.14, *P* = 0.781) or male (ρ = 0.31, *P* = 0.147) dyads. On PR, there was a marginal positive correlation between relatedness and percent home range overlap for female (ρ = 0.75, *P* = 0.064), male (ρ = 0.83, *P* = 0.058), and mixed-sex (ρ = 0.45, *P* = 0.078) dyads.

All sex and age classes of bat-eared foxes dispersed during the study. Of 11 yearling males monitored on both sites (seven Benfontein, four PR), all dispersed before the next breeding season. Of four yearling females monitored on both sites (two Benfontein, two PR), two dispersed, whereas the remaining two bred within their natal range as 2-year olds. Overall, mean dispersal age of yearlings was marginally later (*P* = 0.081) on Benfontein (19.7 ± 5.0 months) than PR (14.2 ± 2.5 months). Overall, six of 12 adult females dispersed, whereas one of four adult males dispersed. Of all foxes that dispersed, significantly (*P* = 0.015) more males (seven of 12) settled <5 km from their initial range compared to females (0 of eight). Final dispersal distances were significantly greater (*P* = 0.011) for females (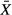 = 19.6 ± 8.0 km, *n* = 4) than for males (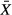 = 3.7 ± 1.0 km, *n* = 8). For males, dispersal distances were greater, but not significantly (*P* = 0.296), on Benfontein (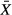 = 4.5 ± 1.4 km, *n* = 5) than PR (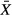 = 2.5 ± 1.0 km, *n* = 3). For females, mean dispersal was 22.7 ± 10.6 km (*n* = 3) on Benfontein, compared to a single dispersal of 10.5 km on PR.

## Discussion

Our analysis revealed both sex and site differences in the spatial genetic structure of bat-eared foxes. These differences were consistent with the effects of female-biased philopatry in the high-density site, and dispersal distance of each sex. As predicted, female dyads exhibited genetic clustering, as neighbors were more related than non neighbors. These results were similar to that found in other canid species that exhibited female-biased philopatry (Ralls et al. 2001; Kitchen et al. 2005). In contrast to our prediction, genetic clustering of female dyads did not occur in the low-density population, where foxes were primarily in monogamous pairs. This suggests that philopatry was not sex-biased in the low-density population, similar to that found in some canid species (Girman et al. 1997; Roemer et al. 2001; Williams et al. 2003). Although the ultimate cause of higher densities of bat-eared foxes on Benfontein was likely higher termite abundance compared to PR (Kamler et al. 2013), our results indicated the larger group sizes on Benfontein resulted from female-biased philopatry. Additionally, delayed dispersal of yearlings on Benfontein also likely contributed to larger group sizes compared to PR. As predicted, male dyads did not exhibit genetic clustering in either population, suggesting that males are less plastic than females in varying their level of philopatry in different populations. Interestingly, our results indicate that genetic clustering and female-biased philopatry can vary within species, and even between closely spaced populations, thus caution should be used when generalizing about the genetic structure of a canid species based on one or a only a few studies.

Overlap of neighboring ranges increased with increasing relatedness for both female and male dyads in the low-density population, which supported our predication. These results were consistent with previous research that showed amount of home range overlap increased with relatedness in other carnivore species (Gompper et al. 1998; Støen et al. 2005; Moyer et al. 2006), including other canid species (Roemer et al. 2001; Kitchen et al. 2005). However, there was no correlation between range overlap and relatedness for either sex in the high-density population on Benfontein. These results seem somewhat counterintuitive because mean overlap of home ranges was greater on Benfontein than PR for both sexes, although significantly so only for females. Thus, one might expect that in areas with greater overlap, foxes would be more tolerant toward relatives, however, this was not the case. Our results could be explained by considering the primary reason for the larger group sizes and home ranges on Benfontein, which was due to predation by jackals (Kamler et al. 2013). On Benfontein, larger groups of bat-eared foxes had better protection from predation (Kamler et al. 2012), however, the larger groups had to forage for food over larger areas, thereby causing the sizes and overlap of home ranges to increase compared to PR. Thus, foxes on Benfontein were likely more tolerant of neighboring groups because more foxes resulted in increased protection against predation from jackals, regardless of relatedness among the foxes, and groups had to roam over large areas. In fact, bat-eared foxes form groups to mob potential predators (Malcolm 1986; Kamler et al. 2012), and that foxes, presumably from different families, formed larger groups to mob larger predators (Maas and Macdonald 2004). Thus, in the absence of the threat of jackal predation on PR, the overlap of home ranges decreased, and foxes from both sexes became more tolerant only toward neighboring relatives of the same sex.

At a broad scale, proportion of relatives decreased as a function of distance, as predicted, however, results were specific to site and sex. For female dyads, distance was correlated with relatedness on Benfontein but not PR. For male dyads, in contrast, distance was correlated with relatedness on PR but not Benfontein. For females, the broadscale genetic structure was consistent with the fine scale genetic structure on both sites, and suggests female philopatry at the high-density site influenced genetic structure at both spatial scales. These results were consistent with previous studies on carnivores that showed genetic relatedness was negative related to geographic distance, at least for certain sexes (Ratnayeke et al. 2002; Spong et al. 2002; Kitchen et al. 2005; Støen et al. 2005; Moyer et al. 2006; Costello et al. 2008). Nevertheless, at the low-density site in this study, lack of female philopatry and the tendencies for females to disperse long distances, resulted in a lack of genetic structure at both spatial scales.

For males, lack of philopatry resulted in lack of genetic structure at the fine scale on both sites. Nevertheless, at the low-density site, males exhibited genetic structure at the broad scale, and this likely was due to their tendency to disperse shorter distances than females. No males bred within their natal range on both sites, however, 58% settled <5 km from their initial range. This is in contrast to females which sometimes bred within their natal range, but otherwise did not settle <5 km from their initial range if they dispersed. Interestingly, the shorter dispersal distances of males appeared to result from their tendency to “float” in areas surrounding their natal range after dispersal (78% of dispersing males), usually accompanied by at least one additional young male (confirmed in seven cases), during which they did not exhibit stable territories. Floating may be advantageous for young male bat-eared foxes, as this may allow them to engage in extra pair copulations before finding a long-term mate (Wright et al. 2010). In contrast, females tended to exhibit solitary straight-line dispersals (88% of dispersing females). These two modes of dispersal have been previously reported in carnivores (Kamler et al. 2000), including other fox species (Macdonald and Courtenay 1996; Kamler et al. 2004a). Although sample size was too low for significance, males dispersed farther, on average, on Benfontein than PR. We speculate that the greater dispersal distance of males on Benfontein was due to the greater density of foxes there, which forced floating males to disperse farther before finding a vacant territory. This hypothesis also explains why there was a genetic structure for males at the broad scale on PR, but not on Benfontein (i.e., farther dispersals in all directions would tend to negate any genetic structure). Similarly, male black bears decreased their dispersal distance in a lower density population, likely in response to reduced intrasexual competition (Costello et al. 2008). However, future research is needed to test this hypothesis for bat-eared foxes.

Our results suggested that movements of not only females and males, but also adults and yearlings, likely assisted with inbreeding avoidance. For example, adult females frequently dispersed (i.e., 50% of all adult females dispersed), possibly to avoid competition with their offspring over local resources. Adult males also dispersed, which would reduce opportunities for them to breed with their philopatric daughters. Dispersing young males tended to float around nearby areas, and often settled in neighboring or twice-removed ranges. In contrast, dispersing females traveled relatively far in a straight-line fashion, thereby avoiding the potential to breed with any male offspring that may have settled in nearby ranges. In fact, long-range dispersals may be an important mechanism of inbreeding avoidance (Gandon 1999). That female bat-eared foxes dispersed farther than males is unusual among carnivores, especially canids, as males typically disperse farther than females in other canid species (Lehman et al. 1992; Gooselink et al. 2010). Additionally, although frequent dispersal of adult males was reported in other fox species (Kamler et al. 2004a,b), the frequent dispersal of adult females is unusual among canids in particular, and mammals in general. The dispersal patterns of bat-eared foxes may be related to their unique evolutionary lineage (Maas and Macdonald 2004). For example, bat-eared foxes diverged from other modern canids up to 12 million years ago, and consequently they have a comparatively long and distinct evolutionary history which includes unique adaptations of up to four sets of extra molar (unique among heterodont placental mammals) and lack of carnassial shearing teeth (unique among terrestrial carnivores), both of which assist with their dietary specialization on termites (Maas and Macdonald 2004; Klare et al. 2011). Bat-eared foxes also are unique among small (<5 kg) canids in that they are group living and not highly territorial, which may have necessitated the need for adult females to disperse, either to reduce competition with offspring or avoid inbreeding. Inbreeding avoidance mechanisms might be especially important in bat-eared foxes, because in contrast to other canid species, frequent mountings of parent–offspring were commonly observed and did not appear to be avoided if opportunities existed (Maas and Macdonald 2004).

In summary, our results showed that female philopatry resulted in both fine and broadscale genetic structuring of females in the high-density population, whereas short-range dispersal of males resulted in broadscale genetic structuring of males in the low-density population. We conclude that a combination of male-biased dispersal rates, adult dispersals, and sex-biased dispersal distances likely helped to facilitate inbreeding avoidance in this evolutionarily unique species of Canidae.

## Data Accessibility

Both microsatellite genotype data for individuals (in GenAlEx format) and microsatellite analyses comparing between individuals are available via DRYAD: doi:10.5061/dryad.85066.

## Supporting Information

**Figure A1.** Distribution of simulations of related and unrelated individuals (*n* = 1000 each) of bat-eared foxes (*Otocyon megalotis*) monitored on two study sites in South Africa.

## References

[b1] Carmichael LE, Nagy JA, Larter NC, Strobeck C (2001). Prey specialization may influence patterns of gene flow in wolves of the Canadian Northwest. Mol. Ecol.

[b2] Costello CM, Creel SR, Kalinowski ST, Vu NV, Quigley HB (2008). Sex-biased natal dispersal and inbreeding avoidance in American black bears as revealed by spatial genetic analyses. Mol. Ecol.

[b3] Creel S, Creel NM (2002). The African wild dog: behavior, ecology, and conservation.

[b4] Dobson FS, Jones WT (1985). Multiple causes of dispersal. Am. Nat.

[b5] Dugdale HL, Macdonald DW, Pope LC, Johnson PJ, Burke T (2008). Reproductive skew and relatedness in social groups of European badgers, *Meles meles*. Mol. Ecol.

[b6] Gandon S (1999). Kin competition, the cost of inbreeding and the evolution of dispersal. J. Theor. Biol.

[b7] Gannon WL, Sikes RS, The Animal Care and Use Committee of the American Society of Mammologists (2007). Guidelines of the American Society of Mammalogists for the use of wild mammals in research. J. Mammal.

[b8] Girman DJ, Mills MGL, Geffen E, Wayne RK (1997). A molecular genetic analysis of social structure, dispersal, and interpack relationships of the African wild dog (*Lycaon pictus*. Behav. Ecol. Sociobiol.

[b9] Goldstein D, Roemer G, Smith D, Reich D, Bergman A, Wayne R (1999). The use of microsatellite variation to infer population structure and demographic history in a natural model system. Genetics.

[b10] Gompper ME, Gittleman JL, Wayne RK (1998). Dispersal, philopatry, and genetic relatedness in a social carnivore: comparing males and females. Mol. Ecol.

[b11] Gooselink TE, Piccolo KA, Warner TR, Van Deelen RE, Mankin PC (2010). Natal dispersal and philopatry of red foxes in urban and agricultural areas of Illinois. J. Wildl. Manag.

[b12] Guyon R, Lorentzen TD, Hitte C, Kim L, Cadieu E, Parker HG (2003). A 1-Mb resolution radiation hybrid map of the canine genome. Proc. Natl Acad. Sci. USA.

[b13] Janecka JE, Blankenship TL, Hirth DH, Tewes ME, Kilpatrick CW, Grassman LI (2006). Kinship and social structure of bobcats (*Lynx rufus*) inferred from microsatellite and radio-telemetry data. J. Zool. (Lond.).

[b14] Ji W, Sarre SD, Aitken N, Hankin RKS, Clout MN (2001). Sex-biased dispersal and a density-independent mating system in the Australian brushtail possum, as revealed by minisatellite DNA profiling. Mol. Ecol.

[b15] Kamler JF, Macdonald DW (2006). Longevity of a wild bat-eared fox. S. Afr. J. Wildl. Res.

[b16] Kamler JF, Gipson PS, Snyder TR (2000). Dispersal characteristics of young bobcats from northeastern Kansas. Southwest. Nat.

[b17] Kamler JF, Ballard WB, Gese EM, Harrison RL, Karki SM (2004a). Dispersal characteristics of swift foxes. Can. J. Zool.

[b18] Kamler JF, Ballard WB, Gese EM, Harrison RL, Karki SM, Mote K (2004b). Adult male emigration and a female-based social organisation in swift foxes, *Vulpes velox*. Anim. Behav.

[b19] Kamler JF, Stenkewitz U, Klare U, Jacobsen NF, Macdonald DW (2012). Resource partitioning among cape foxes, bat-eared foxes, and black-backed jackals in South Africa. J. Wildl. Manag.

[b20] Kamler JF, Stenkewitz U, Macdonald DW (2013). Lethal and sublethal effects of black-backed jackals on cape foxes and bat-eared foxes. J. Mammal.

[b21] Kitchen AM, Gese EM, Waits LP, Karki SM, Schauster ER (2005). Genetic and spatial structure within a swift fox population. J. Anim. Ecol.

[b22] Klare U, Kamler JF, Macdonald DW (2011). The bat-eared fox: a dietary specialist?. Mammal. Biol.

[b23] Kleiman DG, Eisenberg JF (1973). Comparisons of canid and felid social systems from an evolutionary perspective. Anim. Behav.

[b24] Koop K, Velimirov B (1982). Field observations on activity and feeding of bat-eared foxes (*Otocyon megalotis*) at Nxai Pan, Botswana. Afr. J. Ecol.

[b25] Lamprecht J (1979). Field observations on the behaviour and social system of the bat-eared fox *Otocyon megalotis*. Z. Tierpsychol.

[b26] Lehman N, Clarkson P, Mech LD, Meier TJ, Wayne RK (1992). A study of the genetic relationships within and among wolf packs using DNA fingerprinting and mitochondrial DNA. Behav. Ecol. Sociobiol.

[b27] Loison A, Jullien J-M, Meano P (1999). Subpopulation structure and dispersal in two populations of chamois. J. Mammal.

[b28] Maas B, Macdonald DW, Macdonad DW, Sillero-Zubiri C (2004). Bat-eared foxes ‘insectivory’ and luck: lessons from an extreme canid. The biology and conservation of wild canids.

[b29] Macdonald DW, Courtenay O (1996). Enduring social relationships in a population of crab-eating zorros, *Cerdocyon thous*, in Amazonian Brazil (Carnivora, Canidae). J. Zool. (Lond.).

[b30] Maher CR (2009). Genetic relatedness and space use in a behaviorally flexible species of marmot, the woodchuck (*Marmota monax*. Behav. Ecol. Sociobiol.

[b31] Malcolm JR (1986). Socio-ecology of bat-eared foxes (*Otocyon megalotis*. J. Zool. (Lond.).

[b32] Mantel NA (1967). The detection of disease clustering and a generalized regression approach. Cancer Res.

[b33] Moore J, Ali R (1984). Are dispersal and inbreeding avoidance related?. Anim. Behav.

[b34] Moyer MA, McCown JW, Eason TH, Oli MK (2006). Does genetic relatedness influence space use pattern? A test on Florida black bears. J. Mammal.

[b35] Nel JAJ (1993). The bat-eared fox: a prime candidate for rabies vector?. Ondersteport J. Vet. Res.

[b36] Nel JAJ, Mills MGL, Van Aarde RJ (1984). Fluctuating group size in bat-eared foxes (*Otocyon m. megalotis*) in the south-western Kalahari. J. Zool. (Lond.).

[b37] Ortego J, Calabuig G, Aparicio JM, Cordero PJ (2008). Genetic consequences of natal dispersal in the colonial lesser kestrel. Mol. Ecol.

[b38] Peakall R, Smouse PE (2006). GENALEX 6: genetic analysis in Excel. Population genetic software for teaching and research. Mol. Ecol. Notes.

[b39] Pusey AE (1987). Sex-biased dispersal and inbreeding avoidance in birds and mammals. Trends Ecol. Evol.

[b40] Queller DC, Goodnight KF (1989). Estimating relatedness using genetic markers. Evolution.

[b41] Ralls K, Pilgrim KL, White PJ, Paxinos EE, Schwartz MK, Fleischer RC (2001). Kinship, social relationships, and den sharing in kit foxes. J. Mammal.

[b42] Randall DA, Pollinger JP, Wayne RK, Tallents LA, Johnson PJ, Macdonald DW (2007). Inbreeding is reduced by female-biased dispersal and mating behavior in Ethiopian wolves. Behav. Ecol.

[b43] Ratnayeke S, Tuskan GA, Pelton MR (2002). Genetic relatedness and female spatial organization in a solitary carnivore, the raccoon, *Procyon lotor*. Mol. Ecol.

[b44] Raymond M, Rousset F (1995). GENEPOP (version 1.2): population genetics software for exact tests and ecumenicism. J. Hered.

[b45] Roemer GW, Smith DA, Garcelon DK, Wayne RK (2001). The behavioural ecology of the island fox. J. Zool. (Lond.).

[b46] Rousset F (2008). Genepop'007: a complete reimplementation of the Genepop software for Windows and Linux. Mol. Ecol. Resour.

[b47] Sacks BN, Mitchell BR, Williams CL, Ernest HB (2005). Coyote movements and social structure along a cryptic population genetic subdivision. Mol. Ecol.

[b48] Smithers RHN (1971). The mammals of Botswana.

[b49] Spong G, Creel S (2001). Deriving dispersal distances from genetic data. Proc. R. Soc. Lond. B.

[b50] Spong G, Stone J, Creel S, Björklund M (2002). Genetic structure of lions (*Panthera leo* L.) in the Selous Game Reserve: implications for the evolution of sociality. J. Evol. Biol.

[b51] Støen OG, Bellemain E, Sæbø S, Swenson JE (2005). Kin-related spatial structure in brown bears *Ursus arctos*. Behav. Ecol. Sociobiol.

[b52] Temple HJ, Hoffman JI, Amos W (2006). Dispersal, philopatry and intergroup relatedness: fine-scale genetic structure in the white-breasted thrasher, *Ramphocinclus brachyurus*. Mol. Ecol.

[b53] Van Horn RC, Engh AL, Scribner KT, Funk SM, Holekamp KE (2004). Behavioural structuring of relatedness in the spotted-hyena (*Crocuta crocuta*) suggests direct fitness benefits of clan-level cooperation. Mol. Ecol.

[b54] Wagner AP, Creel S, Frank LG, Kalinowski ST (2007). Patterns of relatedness and parentage in an asocial, polyandrous striped hyena population. Mol. Ecol.

[b55] Widdig A, Nürnberg P, Krawczak M, Streich W, Bercovitch F (2001). Paternal relatedness and age proximity regulate social relationships among adult female rhesus macaques. Proc. Natl. Acad. Sci. USA.

[b56] Williams CL, Blejwas KM, Johnston JJ, Jaeger MM (2003). Temporal genetic variation in a coyote (*Canis latrans*) population experiencing high turnover. J. Mammal.

[b57] Wolff JO (1994). More on juvenile dispersal in mammals. Oikos.

[b58] Wright HWY, Gray MM, Wayne RK, Woodroffe RB (2010). Mating tactics and paternity in a socially monogamous canid, the bat-eared fox (*Otocyon megalotis*. J. Mammal.

