# Supplementary figures and images for: Genetic structure, spatial organization, and dispersal in two populations of bat-eared foxes
Source: Ecol Evol. 2013 Jul 26;3(9):2892–902. doi: 10.1002/ece3.683 (PMC3790538; doi:10.1002/ece3.683)

**(A) Simulation Distribution: Benfontein Game Farm**

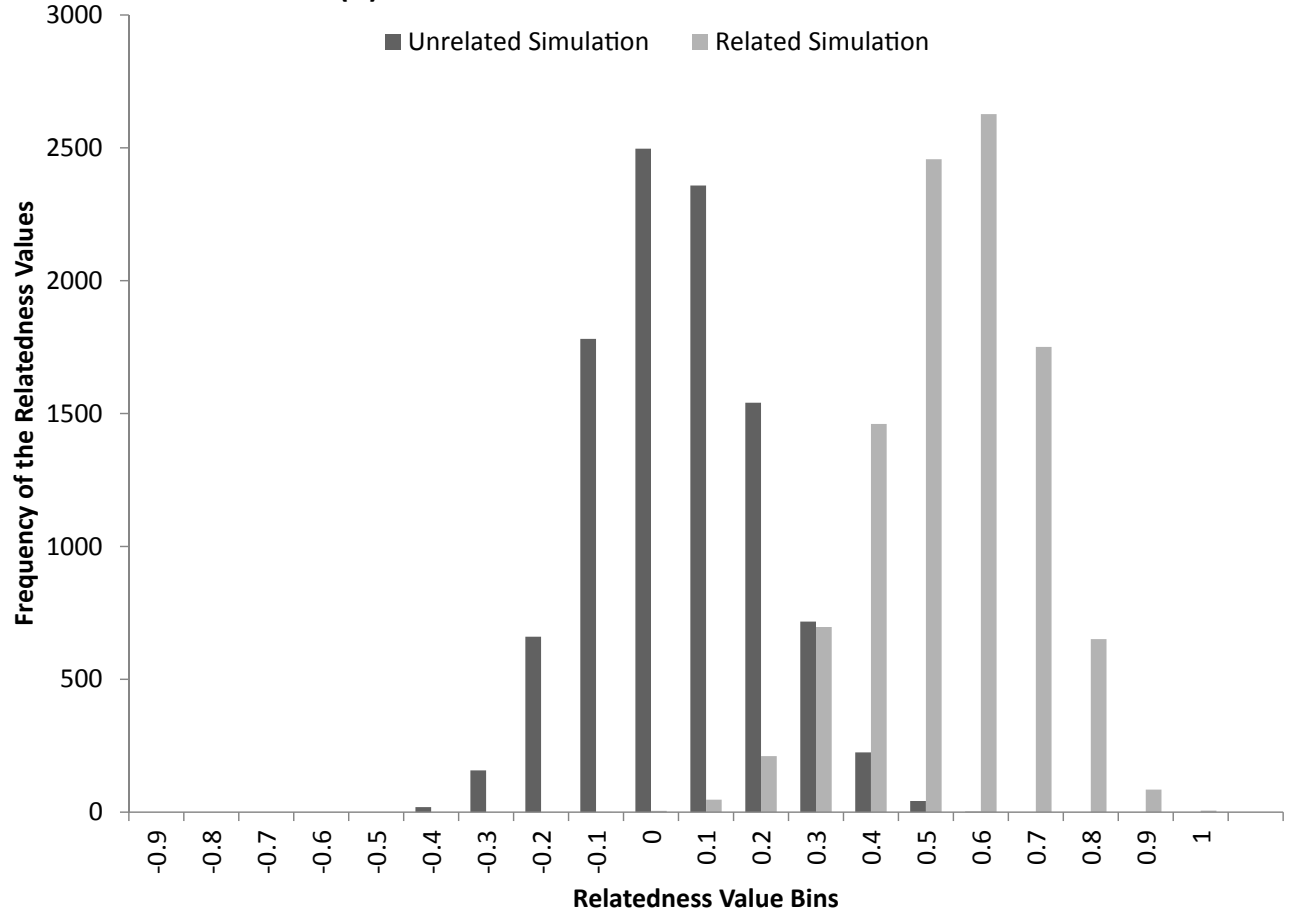

**(B) Simulation Distribution: Private Ranch**

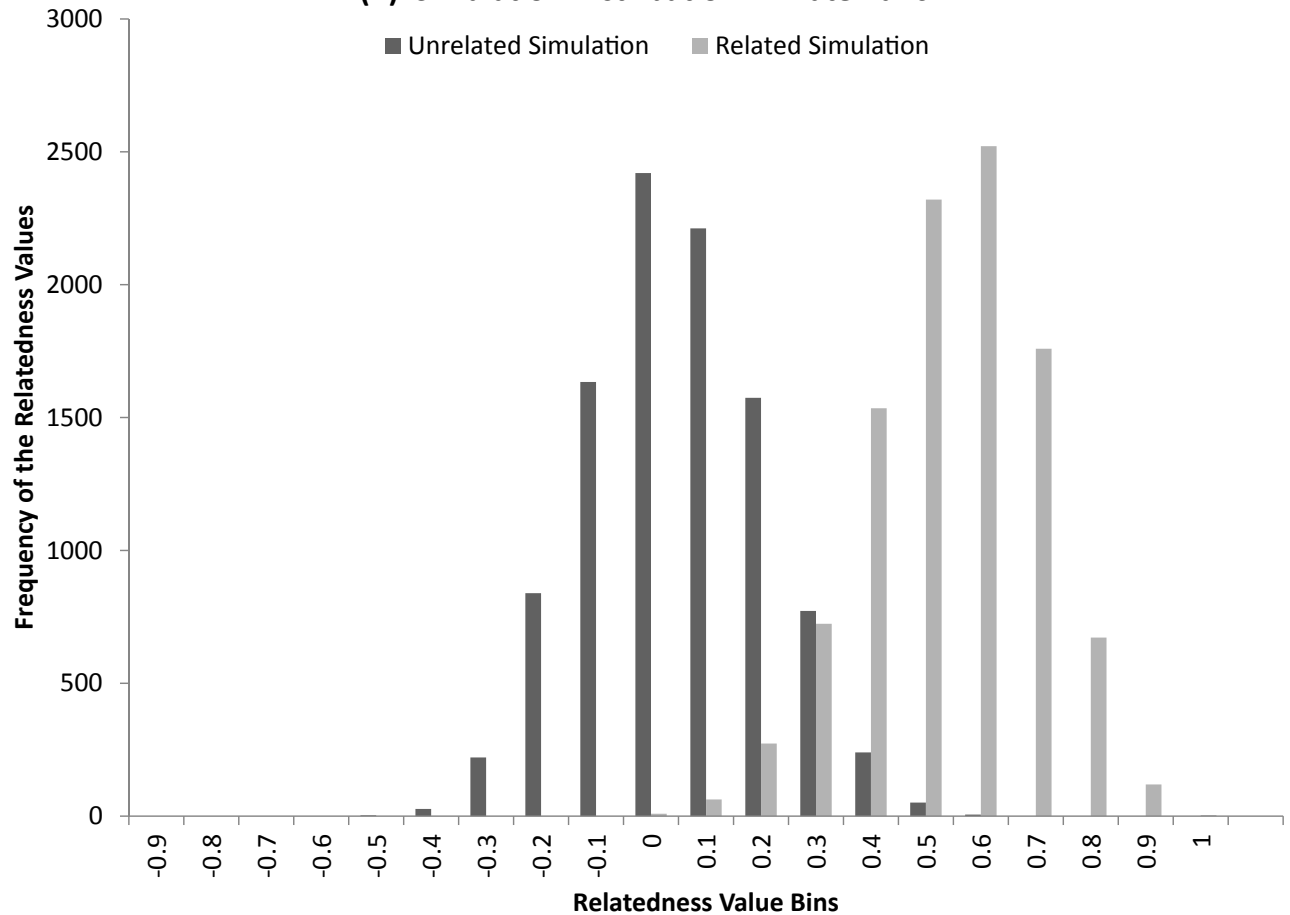

Supplement: Supplementary file 1 [file ece30003-2892-SD1.pdf]
